# Learning fast and accurate absolute pitch judgment in adulthood

**DOI:** 10.3758/s13423-024-02620-2

**Published:** 2025-02-12

**Authors:** Yetta Kwailing Wong, Leo Y. T. Cheung, Vince S. H. Ngan, Alan C.-N. Wong

**Affiliations:** 1 School of Psychology, University of Surrey, 43AC05, Lewis Carroll Building, Guildford, Surrey, GU2 7XH UK; 2 Institute for Sustainability, University of Surrey, Guildford, UK; 3 Department of Educational Psychology, The Chinese University of Hong Kong, Ma Liu Shui, Hong Kong; 4 Department of Psychology, The Chinese University of Hong Kong, Ma Liu Shui, Hong Kong; 5 Surrey Institute for People-Centered AI, University of Surrey, Guildford, UK

**Keywords:** Music cognition, Sound recognition, Perceptual learning, Perceptual expertise, Critical period

## Abstract

Absolute pitch (AP) refers to the ability to identify the pitch of a tone without external references. It is commonly believed that only individuals with special genetic makeup and early musical training within the critical period can develop AP. Recent studies have begun to challenge the critical period notion by showing the possibility of AP acquisition in adults. However, the learning effects could be attributed to learning of pitch height instead of chroma, extended working memory, relative pitch strategies, chance under repeated attempts, pre-existing AP abilities and/or specific cognitive profiles. An 8-week online computerized training program was designed to address these concerns and clarify learnability of AP in adulthood. Twelve musicians on average spent 21.4 h completing 15,327 training trials. By the end of the training, they learned to name an average of 7.08 pitches (ranging from 3 to 12) at an accuracy of 90% or above and within a response-time (RT) window of 1,305–2,028 ms. After training, pitch-naming accuracy was significantly improved by 128.1% (from .139 to .317) and size of error reduced by 42.7% (from 2.62 to 1.50 semitones) for the trained timbre, which generalized partially to an untrained timbre. Overall, results provide more convincing evidence for the learnability of AP judgment in adulthood beyond the critical period, similar to most perceptual and cognitive abilities.

**Supplementary information:**

The online version contains supplementary material available at 10.3758/s13423-024-02620-2.

## Introduction

Absolute pitch (AP) refers to the ability to identify the pitch name of a tone that is presented in isolation. In Western music, AP involves identifying a correct pitch name out of 12 possible answers. As straightforward as it appears, AP is rather rare, with a suggested occurrence ranging from 1 in 10,000 in the population (Takeuchi & Hulse, 1993) to 7–50% among well-trained musicians in music conservatories (Deutsch et al., 2006; Miyazaki et al., 2012). Why is AP so rarely found given the relatively small number of possible alternatives and musicians’ huge amount of exposure to these tones and pitch names?

It is widely endorsed that the development of AP requires a special genetic predisposition and an early onset of musical training during the critical period (Bachem, 1940; Baharloo et al., 1998; Chin, 2003; Deutsch, 2019; Drayna, 2007; Levitin & Rogers, 2005; Ross et al., 2005; Trainor, 2005; Zatorre, 2003). Support of the genetic contribution comes from the observation that AP runs in families (Baharloo et al., 1998, 2000; Drayna, 2007; Gregersen et al., 1999, 2001). Evidence for the critical period comes from the observation that AP possessors tend to start their musical training early in life at or before 5 years of age (Gregersen et al., 1999). Importantly, while learning AP in childhood is possible with months or years of practice (Crozier, 1997; Miyazaki & Ogawa, 2006; Sakakibara, 2014), it is considered impossible in adulthood with no “convincing success observed” in repeated attempts in the past decades (p.434, Bachem, 1940; p.29, Levitin & Rogers, 2005; p.358, Takeuchi & Hulse, 1993).

Recent findings begin to challenge whether the critical period indeed constrains the development of AP. Extensive perceptual training lasting for 12–40 h has been shown to improve pitch-naming performance for most adult participants, who learned to name on average 7.2–9.4 pitches out of 12 across experiments (Wong et al., 2020a, 2020b; Wong et al., 2020a, 2020b; see also Van Hedger et al., 2019). The highest achievers can name 12 pitches at 90% accuracy or above, an average error less than half a semitone, and an average correct RT of around 2 s. This level of performance was comparable to those of real-world AP possessors reported in the literature (see discussion in Wong et al., 2020a, 2020b; Wong et al., 2020a, 2020b). The results suggest that AP is not constrained by the critical period and continues to develop in adulthood.

Despite the previous findings, the learnability of AP judgment in adulthood remains inconclusive because of several methodological issues. First, previous AP training was often limited to one octave (Experiment 2, Wong et al., 2020a, 2020b; Wong et al., 2020a, 2020b) or two octaves (Experiment 1 & 3, Wong et al., 2020a, 2020b). It remains unclear whether the participants really learned the chroma of the tones, which is shared by notes that are one or more octave(s) apart with the same pitch name and considered the essence of AP (Bachem, 1955; Zatorre, 2003), or they merely learned to name a highly specific set of tones based on pitch height.

Second, many tones were presented with their correct pitch names in the course of training, for example during training trials with feedback, practice trials or sample listening (Wong et al., 2020a, 2020b; Wong et al., 2020a, 2020b). Previous studies used 18–20 s continuous glissando stimuli to provide auditory interference before subsequent testing (Wengenroth et al., 2013; Wong et al., 2020a, 2020b; Wong et al., 2020a, 2020b), which should minimize the use of these tones as external reference for most participants (Peterson & Peterson, 1959; Takeuchi & Hulse, 1993). However, it is possible that the auditory interference was not effective enough for some individuals with extended tonal working memory because of their musical expertise (Talamini et al., 2022).

Third, due to a limited range of pitches used in previous studies, the pitch difference between the tones in adjacent testing trials was relatively small (Wong et al., 2020a, 2020b; Wong et al., 2020a, 2020b). This made it easier to use relative pitch strategies during AP tests (Deutsch, 1972; Deutsch & Boulanger, 1984). Specifically, participants could have learned to name a couple of the pitches in an absolute manner, and used them as internally generated references to name other pitches. Although no external references were involved, it was inconsistent with the expectation that participants developed AP with *most or all* pitches (Zatorre, 2003).

Fourth, acquisition of AP ability was often demonstrated by successfully passing the last training level that included all 12 pitches (Wong et al., 2020a, 2020b; Wong et al., 2020a, 2020b). Although the performance requirement for the last training level was high, participants could have passed the final level by chance given unlimited attempts. For example, a person with a true ability corresponding to an accuracy level of 70% could have reached 90% accuracy in a block after many attempts due to statistical fluke.

Fifth, the two highest achievers in a previous AP training already attained a very high accuracy (proportion correct = 60–70%) in an AP test before training (Table 2 in Van Hedger et al., 2019). This performance level approached the average performance of real-world AP possessors in several previous studies (Table 1 in Takeuchi & Hulse, 1993). Also, the two participants reached a performance plateau after the first week of training, and the near-perfect accuracies stayed similar in the following 7 weeks (Fig. 3A; Van Hedger et al., 2019). It is therefore possible that the AP abilities observed at post-test were existing to a large degree before training.

Lastly, one of the training studies selected participants with high auditory working memory (Van Hedger et al., 2019), which makes it unclear whether the observed AP learning could be generalized to the general population. Overall, these issues lead to the question about the quality and consistency of the acquired AP.

**Fig. 1:**
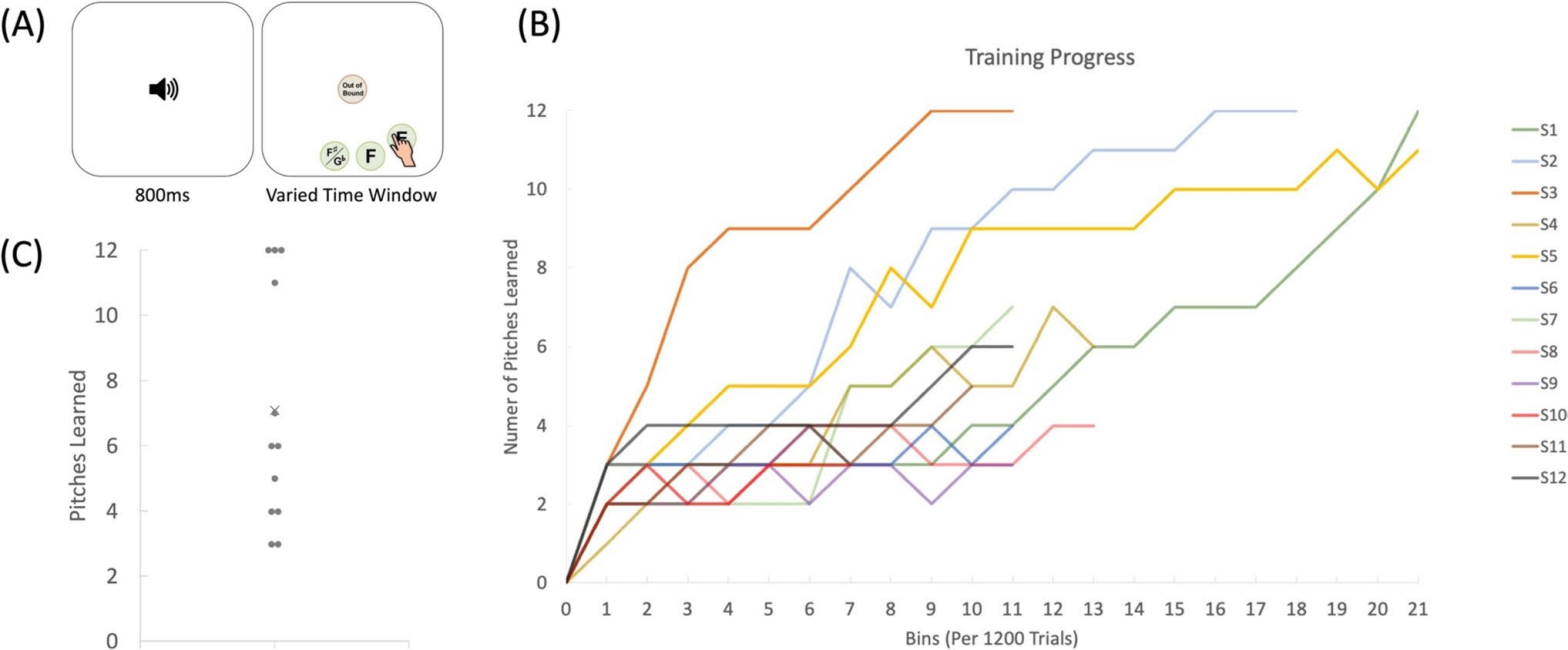
(**A**) An example training trial of the absolute pitch (AP) training, for a level with three pitch names being learned. (**B**) Individual progress of the AP training. Number of pitches learned was defined by the number of pitches that the participants could name with an accuracy of at least 90%, without trial-by-trial feedback or external reference provided, and within specific time windows (see Online Supplementary Material Table 1). Each line represents the learning progress of a participant. (**C**) The number of pitches learned at the end of training for each participant. Each gray dot represents the achievement of one participant, and the cross indicates the group mean. The labels of each participant (e.g., S1, S2, etc.) match with the ones used in the tables and other figures

The goal of the current study was to examine the learnability of AP judgment in adulthood after addressing the issues described above. Adult musicians participated in an online AP training spanning 8 weeks. During training, they were presented a tone each time and clicked on its pitch name within a specific time window (Fig. 1a). As training progressed, more pitches were included and the time window allowed was gradually reduced until participants attained fast and accurate pitch-naming performance with all the 12 pitches (see *Methods*). During pre-post tests, both the trained timbre and an untrained timbre were introduced to examine the extent of generalization of the AP learning.

The methodological issues discussed above were addressed by the following design. First, tones in three octaves were included in the training to facilitate the learning of chroma instead of simply pitch height. Second, no feedback was provided during the post-test, including practice trials, such that no external reference could have existed to enhance AP performance through working memory. Third, during the pre-post tests, successive testing tones were always more than an octave apart. This, together with the relatively short RT windows of 5 s, reduced the effectiveness of any relative pitch strategy applied internally in one’s mind. Fourth, to complete the training, participants were required to pass the final training level four times in a row, with a waiting time of at least 12 h between the third and the fourth attempts. The stable and consistent performance required here would minimize the probability of one accidentally fulfilling the accuracy criterion for the completion of the training upon repeated attempts. Lastly, the participants were not screened with any cognitive abilities and their AP performance was relatively low before training (proportion of correct trials ranged from 0 to 0.36, mean = 0.139), and therefore their AP learning would be representative and cannot be explained by pre-existing AP abilities comparable to that of real-world AP possessors.

If the development of AP ability is not constrained by the critical period and remains learnable in adulthood, there should be no “glass ceiling” for AP learning up to the performance level comparable to that of real-world AP possessors. In this case, the majority of participants should improve in pitch naming after training, and it should be possible for some participants to develop AP ability approaching or comparable to that of real-world AP possessors. Alternatively, if critical period limits AP development, AP improvement should be limited, and none of the participants should result in an AP ability that approached that of real-world AP possessors.

## Methods

### Pre-registration

This study was pre-registered for tonal language speakers and non-tonal language speakers (see the Open Science Statement for the links). They were pre-registered separately because we had planned to study both groups and had no intention to compare the degree of learning of the two groups. Because of dropout and slower training progress than expected (see details below), we decided to deviate from the pre-registration in terms of dropping one of the planned research questions, combining the data from the tonal and non-tonal language speakers during analyses, the amount of training required to participate in the post-test, and the RT window used (see Online Supplementary Material (OSM) for details). Otherwise, the study was conducted as described in the pre-registration documents.

### Participants

Participants were recruited online through personal network, social media, and university mass emails. They included both tonal and non-tonal language speakers. Tonal language speakers were Cantonese speakers recruited from Hong Kong. Non-tonal language speakers were those who have not learned any tonal languages or lived in any community internationally in which most people spoke a tonal language during the critical period, i.e., before the age of 12 years. Participants might have exposure to tonal languages afterwards, for example, one participant started to learn a tonal language at the age of 25 years. They should be able to understand English instructions, be aged 18–59 years, and were required to have considerable achievement or experience in music, i.e., by passing Grade 6 or above in recognized public exams (e.g., ABRSM, Trinity, etc.) or by extensive experience in music playing or performance. They filled out a questionnaire to report their music training background, their motivation, and their expected time commitment for learning AP. Those who indicated substantial interest in acquiring AP and the willingness to commit the time required for the training were invited to the study. Participation was voluntary without any monetary compensation for their time. All the training and testing sessions were conducted online during the pandemic period. All participants provided informed consent, with all methods (including experimental protocols) approved and performed according to the guideline of the Survey and Behavioral Research Ethics Committee of the Chinese University of Hong Kong in accordance with the Declaration of Helsinki.

The sample size of this study was affected by dropout and slow training progress (Table 1). Specifically, ten withdrew from participation, who had completed 5.06 h of training on average (SD = 3.92; range = 0.83–12.2). Also, despite their strong interests in learning AP, 25 participants could not keep up with the expected time commitment of the training, and completed 3.80 h of training on average (SD = 2.48; range = 0.37–8.10). This was likely caused by the voluntary nature of the study during the pandemic because it did not occur in our previous paid online training study (Wong et al., 2020a, 2020b).

**Table 1:** Number of participants included and excluded in the post-test and the subsequent data analyses. Among the withdrawn participants, their reasons for withdrawal included other commitments (N = 6) and health reasons (N = 1); three participants did not provide any explanations

			Number of participants			
			Tonal	Non-tonal	Subtotal	Total
≥ 10 h of training and post-test participated						13
	Included in subsequent data analyses		7	5	12	
	Excluded (failure to follow instruction)		1	0	1	
Post-test not participated						35
	Withdrawn				10	
		< 1,200 trials completed	0	3		
		≥ 1,200 trials completed	2	5		
	< 10 h of training				25	
		< 1,200 trials completed	3	2		
		≥ 1,200 trials completed	14	6		
Total attempted participants						48

Thirteen participants who completed 10 h of training or more within 8 weeks were invited for post-test. One participant was excluded from data analyses because, during the post-test, she listened to two reference tones on her iPad during the break between two blocks of testing trials even though this was strictly prohibited as explicitly explained in the instruction (see below for details about monitoring measures of participants’ behavior during online testing).

As a result, 12 participants were included in the analyses. They included seven tonal and five non-tonal language speakers (four males, seven females, and one with non-binary gender), and were 27.8 years old on average (range 19–44, SD = 8.58 years; Table 2). They had on average 12.8 years of musical training (SD = 5.42) and spent 5.08 h on musical practice every week (SD = 3.73). This sample size provided > 0.95 power, at *p* = 0.05, to reveal the effect of improved AP ability before and after training based on the training effect size in a previous training study (Cohen’s d = 1.30 and 2.09 for improvement in the proportion of correct trials and in size of pitch-naming error, respectively (Wong et al., 2020a, 2020b). Separate analyses that included only those who had completed the 25 h of training, as originally planned in the pre-registration, led to similar results in the ANOVA analyses (see OSM).

**Table 2:** Language and music training background of the participants. The data of the onset of music training of tonal language speakers were missing due to procedural error

Participant number	Age	Tonal/non-tonal speakers	First language	Other languages	Highest music exam passed	Duration music training (years)	Major instrument(s)	Onset music training (years old)	Number of Pitch Learned
S1	34	Non-tonal	Hebrew	Russian, English, French	/	4	Piano	24	12
S2	19	Tonal	Cantonese	English, Mandarin	Trinity ATCL	8	Violin		12
S3	38	Non-tonal	German	English, Cantonese, Mandarin	Central Conservatory of Music, China, Grade 9	17	Piano, Violin, Sheng	7	12
S4	19	Tonal	Cantonese	English, Spanish	Piano ABRSM Grade 8; Violin ABRSM Grade 6	14	Piano, Violin		6
S5	44	Non-tonal	Hebrew	English	/	20	Piano	16	11
S6	19	Tonal	Cantonese	Mandarin, English, Korean	ABRSM Grade 6	8	Piano		4
S7	24	Non-tonal	English	French, German, Italian	/	12	Flute	9	7
S8	22	Tonal	Cantonese	English, Mandarin, Japanese, Korean	ABRSM Grade8	17	Violin		4
S9	23	Non-tonal	English	Spanish	/	7	Voice	9	3
S10	23	Tonal	Cantonese	English	ABRSM Grade 8	10	Piano		3
S11	35	Tonal	Cantonese	/	Trinity ATCL	20	Piano		5
S12	33	Tonal	Cantonese	/	ABRSM Grade 8	17	Piano		6

**Fig. 2:**
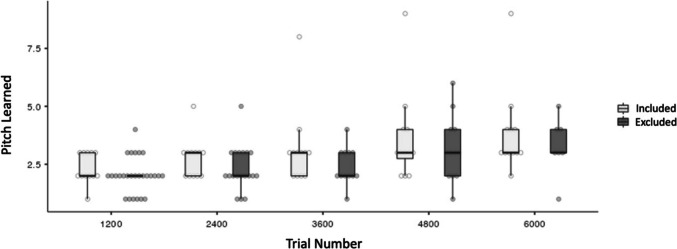
Boxplot of the progress of the absolute pitch (AP) training for participants who were included or excluded from the analyses (Table 1). Number of pitches learned was defined by the number of pitches that the participants could name with an accuracy of at least 90%, without trial-by-trial feedback or external reference provided, and within the specific time windows (Online Supplementary Material Table 1)

It is possible that the excluded participants were those who learned AP less effectively, and therefore the results reported in this study would have over-estimated the AP learning in the population. To test this possibility, the learning progress was compared between the included and excluded participants during the first 6,000 trials of learning (Fig. 2). Mann–Whitney U tests were used to test whether the number of pitches learned between the included and excluded participants during each of the first five bins of training (1,200 trials per bin, corresponding to roughly an hour of learning) were similar. Mann–Whitney U tests were used because Shapiro–Wilk tests showed a violation of the normality assumption for all of the bins (*p*s ≤ 0.003). Those who completed less than 1,200 trials over 8 weeks were not considered in this analysis (N = 8; mean = 735.5 trials; SD = 316.1; Table 1). The participant who failed to adhere to the instruction of not using an external pitch reference during testing was not considered in this analysis because this exclusion was not relevant to the potential differences in learning progress of the dropouts.

Results showed that there were no observable differences in all of the bins between the two groups (all *p*s > 0.21). Bayes statistics showed weak evidence supporting no difference in the number of pitches learned in all bins (Bayes Factor_10_ ranged between 0.337 and 0.624). Similar analyses were not performed from the sixth bin onwards as there were only four or less participants left in the excluded group. These showed that the risk of over-estimating the AP learning by investigating the included participants only was minimal.

### Materials

Piano tones from three octaves (C4 to B6) were generated using two different digital pianos (Roland FP60 and Yamaha Arius), and guitar tones spanning the same range were generated by an online synthesizer. The tones were presented in clips of 800 ms in length, with a 100-ms ramp at the beginning and at the end using Audacity. The tones were then normalized to the sound pressure of 0.99 Pascal in volume using Praat. The training used piano tones, while the pre-post test included piano tones to examine the training effect and guitar tones to examine learning generalization to an untrained timbre. The piano tones used during training and pre-post tests were generated from different sources (Roland FP60 for training and Yamaha Arius for testing) so as to ensure that the observed learning effects were general to the timbre of piano instead of a specific acoustic spectrum. Participants were recommended to use a headphone during training and were required to use a headphone during pre-post tests (see below).

### Training

For AP training, participants were instructed to finish 25 h of training online over 8 weeks, and complete at least 2 h of training per week. The training included 288 training levels, with 24 levels for each additional pitch, with the goal of training participants to acquire fast and accurate AP judgment while recognizing small improvements in the process.

Training started with one pitch (the pitch “F”) with 24 training levels that require a progressively higher level of accuracy to pass (from 20 to 90%), with feedback provided at first and followed by no-feedback levels, and with progressively shorter RT windows (from 1,183 ms for learning one pitch to 2,028 ms for learning 12 pitches). When one passed the 24th level, a new pitch adjacent to the original set of pitch(es) would be added to the training followed by another 24 levels of training. The new pitch would be alternatively taken from the upper or the lower side of the starting pitch. For example, “E” was added when one passed all levels with “F,” and then “F#” was the pitch added next. This cycle repeated until all 12 pitches were introduced.

To ensure that participants were learning the pitch names instead of working on a relative judgment of highest versus lowest tones within the training set, four additional pitches were included in the training around the learning pitches, i.e., + 2, + 1 semitones relative to the highest pitch being trained, and −1, −2 semitones relative to the lowest pitch being trained. Participants should indicate that these four pitches were “out of bound,” i.e., tones that did not match any of the to-be-learned pitch names. In other words, when participants were learning “one pitch,” they were in fact hearing tones of five pitches. They were required to name one of the pitches and identify the other four as “out of bound.” The out-of-bound tones faded out when the training set became larger, i.e., when there were no pitches left untrained immediately above or below the training set. They were no longer included when the training set included all 12 pitches.

During training, participants clicked on a button in the middle of the screen to start a trial. Then, a tone would be presented for 800 ms, and a response image with all the possible response options (the training pitch names and an option of “out-of-bound”; Fig. 1a). Participants were required to click on one of the options within the RT window. Responses after the designated RT window would be regarded as errors. Also, semitone errors, i.e., answers that were one semitone off the correct answers, were regarded as errors.

Participants could opt for hearing sample tones before the start of each training level. They could click on one of the trained pitches, and then they would hear all of the tones associated with that pitch (across three octaves) presented in a random order. They could repeatedly listen to the sample tones without time limit until they decided to proceed. If the subsequent level was one without feedback, a Shepard tone, i.e., a continuously descending tone that does not have clear transition between isolated pitches, would be presented for 20 s to disrupt the working memory associated with the heard tone samples.

To make the best use of participants’ training time, we also introduced a level-jumping mechanism. This allowed participants to jump to a higher level that matched with his or her demonstrated performance and skip the training levels in between. For example, although level 1 requires only an accuracy of 20% to pass, if participants attained 90% accuracy at this level, they could skip the following levels that require 30–90% accuracies, and proceed to the level *after* that requires 90% accuracy.

When the number of trained pitches was five or above, two special exercises were additionally provided after every 15 attempted training levels so as to pinpoint individual learning needs. The special exercises focused on a target pitch with the lowest accuracy over the last 15 attempted training levels. In the special exercises, participants were prompted to select whether the presented tone was the target pitch or other out-of-bound pitches. The first special exercise included 12 trials with feedback, and the second special exercise included 22 trials without feedback. Also, when participants attempted the same level for ten times or more without passing it, they were contacted by the experimenter and provided an option to return to a lower level, e.g., those with feedback or with a longer RT, to gradually learn again. This was up to participants to decide and the exact level to go to was discussed on an individual basis. This option was adopted at 1.26% of the total attempted levels.

Other motivational measures were added to gamify the training experience. For example, participants could earn tokens by achieving various accuracies even if they did not pass a training level. With ten tokens, they had a chance of doubling the points earned at a trial such that it would be easier to pass a level. Sometimes these chances of doubling the points could appear randomly (1 in 80 trials on average) as an unexpected reward. These added strategic elements to the training for motivational purposes. Importantly, at the final 24th level of learning each additional pitch, none of these strategic elements were allowed, such that participants needed to achieve the expected performance without extra assistance before moving on. Finally, participants were informed about the number of consecutive days they had been participating in the training. This number would drop to zero if they did not do any training for more than two calendar days. This served as a reward for encouraging consistent training behavior.

Since the training was conducted online, participants could perform a training session anywhere as long as they had access to the Internet. To encourage participants to find a suitable place and time with stable Internet connection to do the training, participants were informed that only training sessions lasted for more than 15 min would be counted towards their 25 h of training time.

To finish the training, participants should either complete 25 h of training or fulfil the following criteria: (1) passing the 288th training level for three consecutive times; and then (2) achieving a “one-take-pass” performance of the 288th (final) level after at least 12 h of no-training waiting time. This ensures that participants completed the training with a consistent performance instead of simply achieving the 90% accuracy criterion once by chance with repeated attempts.

### Pre-post tests

An AP-naming test was used as the pre-post test. Training effects were assessed with a one-group repeated-measures design with two within-subject factors of Pre-post (pre-test/post-test) and Timbre (trained/untrained). No control training group was required here because AP is known to be notoriously difficult to acquire even with decades of musical training and exposure (Takeuchi & Hulse, 1993; Ward, 1999), and therefore placebo effects were not a concern. A previous study also reported that repeated attempts of the same AP test in the same session, using a pitch name verification task and each with 144 trials, resulted in similar performances (d’ = 0.27, 0.42, and 0.30 for the three attempts, respectively; Wong et al., 2020a, 2020b). This demonstrates that AP performance showed limited changes just by repeated testing.

Each pre-post test included 144 trials, involving tones in 12 pitches in two timbres (trained/untrained) and three octaves (octaves 4–6), and each appearing twice. Participants finished a block of trials with the trained timbre, followed by one with the untrained timbre. All the tones of different octaves within each block were randomized with the restriction that tones in adjacent trials were more than 12 semitones apart to discourage the contribution of relative pitch in AP performance (Carroll, 1975; Deutsch, 1972; Miyazaki, 1988, 1989, 1990). Participants were required to respond as fast and as accurately as possible, with a maximum RT window of 5 s. Semitone errors were regarded as errors, and so were responses beyond the 5-s time window.

Since the pre-post tests were conducted online, several measures were taken to prevent the possibility of (or at least identify) cheating behavior, for example, to inappropriately enhance performance by relying on external devices during testing. First, participants were required to use personal headphones, with an additional checking of the audio setup of the computer before testing to ensure that the only audio output was the headphone. This made sure that no other external devices could possibly receive the audio output of the tones during testing to provide any extra hints. Second, before testing, participants were required to show the testing environment (including the surface of their table) to experimenters with a webcam to reduce the likelihood that there would be devices that could be of extra assistance. Third, the test sessions were recorded using Zoom, during which participants were required to share the whole desktop screen with the experimenter. This was to monitor any additional software or web browsers available on the computer. Lastly, participants were required to show their faces during the Zoom recording such that the experimenter could monitor their face and eye movement for any suspicious search of extra hints during testing. These measures discouraged cheating and minimized the possibility that participants received any extra help during the pre-post tests. As mentioned above, one participant was excluded because the Zoom recording showed that she listened to two tones on her iPad during the break between two blocks of trials.

### Analyses

Analyses were performed with Jamovi (v. 2.3.21). As planned, repeated-measures ANOVA with Pre-post (pre-test/post-test) x Timbre (trained/untrained) were conducted on the proportion correct, size of error (i.e., absolute deviation of the response from the correct answers in the unit of semitones), and the correct RT during pre-post tests.

## Results

### Training progress

On average, participants completed 21.4 training hours (SD = 4.18; range 12.6–26.5 h) and 15,327 training trials (SD = 5,293; range 7,500–24,524 trials). At the end of the training, participants were exposed to 11.08 pitches on average (including the “out-of-bound” tones), and were able to name 7.08 pitches (SD = 3.65; range 3–12) with an accuracy of 90% or above, without any feedback or any extra assistance, and within a RT window of 1,305–2,028 ms that varied depending on the number of pitches being trained (Figs. 1B–C; OSM Table 1).

### Pre-post tests

**Fig. 3:**
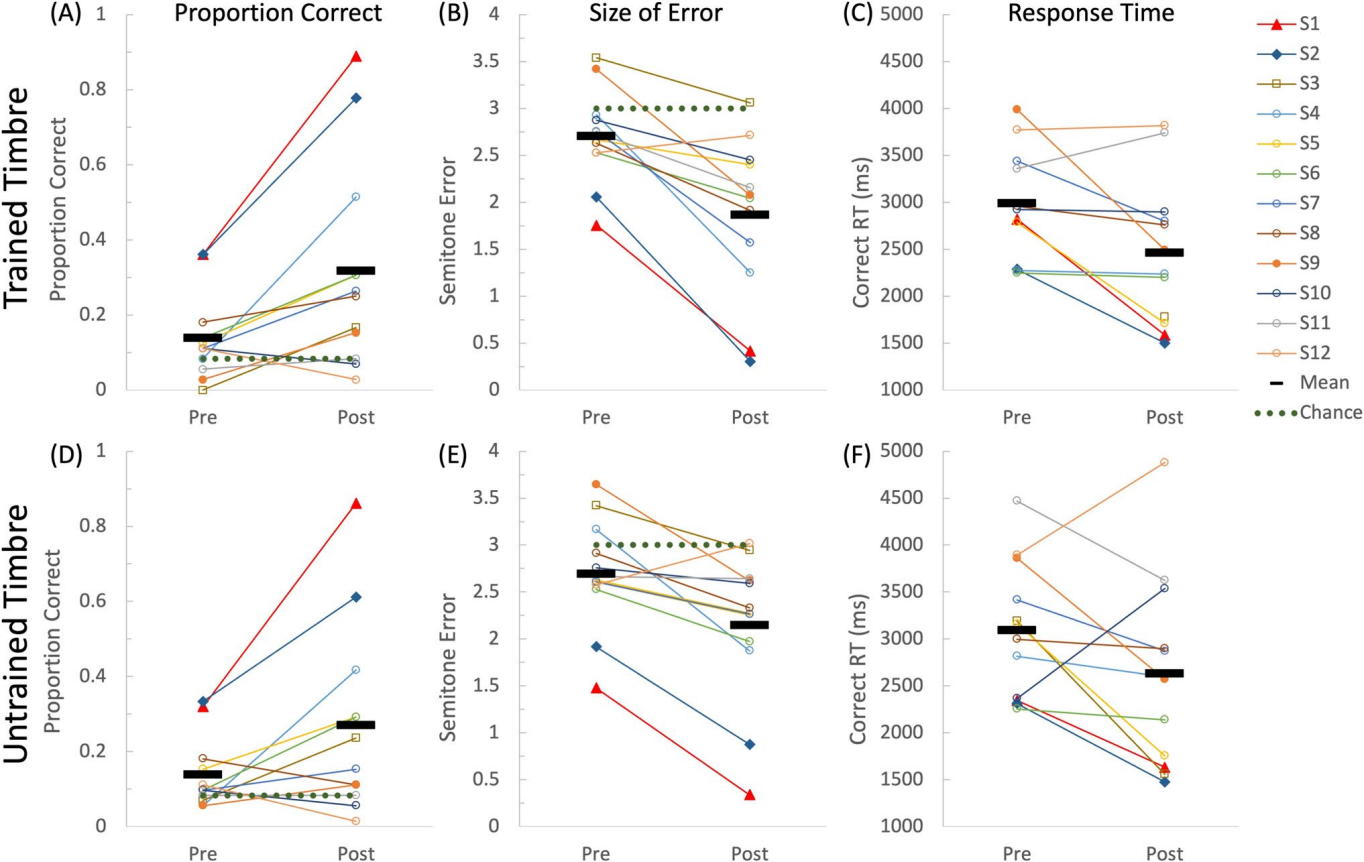
The pre-test and post-test performance for absolute pitch (AP) naming. The top panel shows performance for proportion correct, size of error (absolute deviations in semitone), and correct response time (RT; ms) for the trained timbre for (**A–C**), and the bottom panel shows that for the untrained timbre (**D–F**). The thick black bars show the group mean of each condition. The dotted lines show the chance levels of performance in terms of proportion correct and size of error. The labels of each participant (e.g., S1, S2, etc.) match with the ones used in the tables and other figures

Overall, participants improved in note naming after training as indicated by an increased proportion of correct responses, reduced error, and reduced correct RT, with some generalization to the untrained timbre (Fig. 3). One participant was not included in the RT analyses because no trial was performed correctly during pre-test (Fig. 3).

After training, there was a 128.1% increase^1^ in proportion correct for the trained timbre (mean_pre_ = 0.139; mean_post_ = 0.317), with 10 out of 12 participants showing a numerical improvement. There was also a 42.7% drop in size of error for the trained timbre (mean_pre_ = 2.62 semitones; mean_post_ = 1.50 semitones), with 11 out of 12 participants showing a numerical improvement. A 2 × 2 analysis of variance (ANOVA) with Prepost (pre-test/post-test) and Timbre (trained/untrained) as within-subject factors showed a main effect of Prepost for proportion correct, *F*(1,11) = 8.15, *p* = 0.016, *η*_*p*_^*2*^ = 0.426, and for size of error, *F*(1,11) = 19.7, *p* < 0.001, *η*_*p*_^*2*^ = 0.642, both indicating a higher accuracy after training. The main effect of Prepost was close to significant for correct RT, *F*(1,10) = 4.32, *p* = 0.064, *η*_*p*_^*2*^ = 0.302, with a trend of faster responses after training (mean_pre_ = 3,034 ms; mean_post_ = 2,624 ms). The main effect of Timbre was significant for size of error, *F*(1,11) = 4.97, *p* = 0.048, *η*_*p*_^*2*^ = 0.311, with a smaller error for the trained (mean = 2.28 semitones) than untrained (mean = 2.42 semitones) timbre. The interaction between Prepost and Timbre was significant for proportion correct, *F*(1,11) = 7.94, *p* = 0.017, *η*_*p*_^*2*^ = 0.419, and for size of error, *F*(1,11) = 11.8, *p* = 0.006, *η*_*p*_^*2*^ = 0.517. Post hoc Tukey tests (*p* < 0.05) revealed that proportion correct was higher during post-test than pre-test only for the trained timbre but not for the untrained timbre, while size of error dropped for both timbres but more so for the trained one.

As shown in Fig. 3, two participants (S1 and S2, non-tonal and tonal speakers, respectively) performed considerably better than the other participants during the post-tests for tones in the trained timbre in terms of proportion correct (0.89 and 0.78, respectively), size of error (0.42 and 0.31, semitone respectively), and correct RT (1,586 ms and 1,502 ms, respectively). Results of the above Prepost x Timbre ANOVAs remained similar after excluding these two participants (see OSM), indicating that the group training effects were not driven only by these two participants.

The third participant who completed all training levels showed a low accuracy and a large error during the post-test (S3 with brown square; Fig. 3). In the optional written feedback provided to us, he shared that he tried to listen to the sample tones for reference and then apply the relative pitch strategy to “legally game the training system.” While relative pitch strategies allowed him to get through the AP training in a less controlled environment, the complete absence of reference during the post-test disabled his use of relative pitch strategies (see *Methods*) and therefore revealed that his AP was indeed limited.

## Discussion

An 8-week online computerized training program was designed to address potential methodological issues in previous studies and clarify the learnability of AP in adulthood. By the end of the training, participants were able to name 7.08 pitches (range = 3–12 pitches) at an accuracy of 90% or above within a response-time window of 1,305–2,028 ms. Pre-post tests showed significantly improved pitch-naming performance, with a 128.1% increase in accuracy (mean proportion correct from 0.139 to 0.317) and a 42.7% decrease in size of error (from 2.62 to 1.50 semitones) after training for the trained timbre, which generalized partially to tones of an untrained timbre. Two participants passed all training levels and showed highly accurate and fast AP judgment with all 12 pitches in the post-test (Figs. 1 and 3), demonstrating that there was no “glass ceiling” for AP development in adulthood.

Importantly, the performance attained in the current study cannot be attributed simply to pitch height learning, external references in one’s working memory, luck, or specific cognitive profiles. First, participants learned to attach the same pitch name to all tones sharing the same chroma in three octaves, demonstrating that they learned the chroma of the tones rather than the specific pitch height of individual tones. Without feedback at any point during the post-test, no external reference was available in one’s working memory. For participants who passed all the training levels, accurate and fast AP performance was consistently demonstrated four times in a row and again during the post-test, indicating stable and replicable performance that cannot be explained by luck. The lack of cognitive screening during participant recruitment suggest that the observed AP learning cannot be explained by specific cognitive profiles.

While there is no known strategy to ensure a complete elimination of relative pitch strategies (Wong et al., 2020a, 2020b), the current study adopted stringent measures to minimize their use. This included large pitch distances between adjacent trials, known to make relative pitch strategies more difficult to apply (Baharloo et al., 1998; Carroll, 1975; Deutsch, 1972; Deutsch & Boulanger, 1984; Deutsch et al., 2006; Miyazaki, 1988, 1989, 1990; Van Hedger et al., 2019), and achieving speeded correct responses of 1.5–2 s by the two participants who learned all 12 pitches (OSM Table 1, Fig. 3C). The RT was achieved using arbitrary mouse-click selections among 12 alternatives and was comparable to that of real-world AP possessors (Wong et al., 2020a, 2020b; Wong et al., 2020a, 2020b). Combining these measures minimized the room for internal pitch calculations, making relative pitch strategies even more implausible than previous studies that adopted longer response windows (typically 3 s or more) or more automatized writing responses (e.g., Baharloo et al., 1998; Deutsch et al., 2006; Van Hedger et al., 2019).

The attained AP performance after training is unlikely to be explained by pre-existing abilities. The two participants who acquired accurate and fast AP performance did show higher AP accuracies than others during pretest (proportion correct = 0.36 and 0.36, size of error = 1.75 and 2.06 semitones for S1 and S2 respectively; Fig. 3). However, individuals with such performance were rarely considered “AP possessors” because their accuracy was lower than half of that frequently demonstrated by real-world “AP possessors” (e.g., Takeuchi & Hulse, 1993). Importantly, it took them 18 and 21 h to complete the training with slow and gradual improvement (Fig. 1B). Therefore, it is likely that their AP ability gradually developed during the training.

While the most successful learners in AP training started with relatively better pretest performance in some studies (e.g., the current study; Van Hedger et al., 2019), it was not consistently observed in other training studies (Wong et al., 2020a, 2020b; Wong et al., 2020a, 2020b). For example, one participant who acquired AP had a lower pretest accuracy (0%) than the group mean (13.9%; Wong et al., 2020a, 2020b). Hence there was no evidence suggesting that pretest performance is an important factor in explaining AP training success.

The ability to learn fast and accurate AP judgment in adulthood in all participants, ranging from a few to all 12 pitches, challenges the widely endorsed notion that the critical period constrains the development of AP (Baharloo et al., 1998; Deutsch, 2019; Drayna, 2007; Levitin & Rogers, 2005; Takeuchi & Hulse, 1993; Trainor, 2005; Zatorre, 2003). The non-tonal speaker who attained fast and accurate AP performance after training started music training at 24 years of age (Table 2), indicating that early onset of musical training was not essential for AP development during adulthood.

The current findings are inconsistent with the core assumption of the two-component model of AP (Levitin & Rogers, 2005). This cognitive model explicitly states that most people can perceive tones presented in isolation and form AP memory well (Schellenberg & Trehub, 2003), but only a selected few can overcome the difficulty in pitch labelling, i.e., the ability to associate a verbal label with a pitch. However, all participants in the current study learned to quickly and accurately name at least a subset of the pitches, from 7.08 pitches on average to all 12 pitches in two individuals (Fig. 1C). The continuous distribution found in the current and previous training studies (Miyazaki & Ogawa, 2006; Wong et al., 2020a, 2020b; Wong et al., 2020a, 2020b) shows that, apart from perceiving isolated tones and establishing AP memory, most people *can* associate verbal labels with tones in an absolute manner (see also Van Hedger et al., 2020).

Our findings provided additional evidence that exposure to tonal languages during early childhood is not an essential condition for learning AP judgment in adulthood. Non-tonal language speakers, who have not been exposed to tonal languages before 12 years of age, can acquire AP judgment with tones in a single octave (Wong et al., 2020a, 2020b) and with tones in multiple octaves (participant S1 in the current study). Future studies may directly compare the development of AP among tonal and non-tonal language speakers to test if previous exposure to tonal languages, while unnecessary, leads to any advantages in AP acquisition during training as suggested in previous work (Deutsch et al., 2004, 2006, 2009).

Since the training was performed with piano tones, it was possible that pianists might have enjoyed the advantage of timbre familiarity and therefore had a larger degree of AP improvement. However, the data did not support this possibility. First, the average improvement of the non-pianists was numerically larger than that of pianists for the trained timbre (OSM Table 2). Also, some pianists improved relatively slowly (e.g., S10), while one of the pianists achieved fast and accurate AP (S1). The other participant who achieved fast and accurate AP was a violinist. Overall, there was no evidence supporting that pianists enjoyed an advantage of learning AP due to timbre familiarity.

Some researchers may have concerns about the above interpretations of findings. First, some may question whether the performance of the two highest-achieving participants after training should be regarded as “true” AP. In the literature, “true” AP refers to AP that is highly accurate, fast, effortless, and automatic (Ross et al., 2005; Takeuchi & Hulse, 1993). This is often considered categorically different from other forms of AP, for example, “quasi,” “partial,” “implicit,” and “pseudo” AP, which generally refer to AP with a lower accuracy, limited to a subset of pitches, or expressed in terms of perceptual discriminability rather than explicit labelling (Bachem, 1940; Deutsch, 2002; Takeuchi & Hulse, 1993; Van Hedger et al., 2015; Ward, 1999; but see Bermudez & Zatorre, 2009; Van Hedger et al., 2020). Apart from performance level, it was also reported that some “true” AP possessors can accurately produce, i.e., sing, a tone of a certain pitch absolutely (Takeuchi & Hulse, 1993), and others can better differentiate between tuned and mistuned tones (Ross et al., 2005). Hence, the key question underlying this concern is how “true” AP is defined.

Importantly, the literature does not have any consensus about what performance level “true” AP represents, and many studies simply relied on self-report to categorize participants as “true” AP (see discussion in Bermudez & Zatorre, 2009; Wong et al., 2020a, 2020b). Notably, the two highest-achieving participants in the current study, based on the accuracy and speed of their AP judgment, would be categorized as “true” AP in more than 80% of the published papers that adopted an objective performance-based definition of AP (Wong et al., 2020a, 2020b). Given that it is representative in the literature to consider these two participants “true” AP, researchers who question the appropriateness of this interpretation should explicitly define the alternative level of performance for defining “true” AP, justify why this definition is more appropriate, and explain why the performance observed here should still be considered *categorically* different from such defined “true” AP.

Other researchers might agree that the two highest-achieving participants had a comparable performance with that of “true” AP, but regard them as outliers and therefore their achievements were not representative of the feasible development of AP in the population. This concern might stem from two different reasons. First, from the perspective that AP is dichotomous (Athos et al., 2007), these two participants had above-chance performance during the pretest (proportion correct = 0.36 and 0.36, size of error = 1.75 and 2.06 semitones for S1 and S2, respectively), which did not fit into either the “all” or “none” categories of AP (Fig. 3). Hence, they might simply be outliers and not representative of the population. However, the dichotomous view has been challenged by empirical findings that showed a considerable number of musicians with an above-chance performance similar to that of these two participants but far from that of “true” AP (Bermudez & Zatorre, 2009; Van Hedger et al., 2020; Vanzella & Schellenberg, 2010). Therefore, cases like these should not be disregarded simply because they do not fall into either the “all” or “none” category of AP ability.

Another reason to consider these two participants as outliers comes from the perspective that the critical period represents “a central tendency, an average age at which individuals pass through a particular developmental stage” (p.106, Levitin & Zatorre, 2003). Accordingly, the rare individuals (i.e., outliers) who could learn AP beyond the critical period are well expected purely on statistical ground. Notably though, on average 14.7% of participants (range = 10.0–18.1%), i.e., about one in seven, were able to learn to name all 12 pitches after training (the current study; Wong et al., 2020a, 2020b; Wong et al., 2020a, 2020b). Also, some participants may have been able to name all pitches accurately had the AP training lasted for longer (e.g., those who acquired naming for 10 or 11 pitches). These participants are too many to be considered outliers. Also, the above description of the critical period (Levitin & Zatorre, 2003) deviates from its typical definition that considers early experience *essential* for normal development (Knudsen, 2004). Music experience in early life does relate to a higher tendency of AP development (Gregersen et al., 1999), but this is more consistent with the concept of a “sensitive period,” which states that experience has a particularly strong effect during early life but the psychological function concerned remains plastic throughout the lifespan (Hooks & Chen, 2007; Sengpiel, 2007). Under this definition, acquiring “true” AP in adulthood should well be possible, albeit more difficult. Adult AP training studies like the current one (Wong et al., 2020a, 2020b; Wong et al., 2020a, 2020b) often involved a much shorter training period than those with young children. For example, children took 6 weeks to learn AP for one pitch (Crozier, 1997), and they took 1.5–3 years to learn all pitches (Miyazaki & Ogawa, 2006; Sakakibara, 2014). Whether a sensitive period of AP development exists should be clarified by matched methods and measurements of AP learning between young children and adults.

## Supplementary information

Below is the link to the electronic supplementary material.

Supplementary file1 (DOCX 28 KB)

## Data Availability

The datasets analyzed in the current study are available on the Open Science Framework repository at: https://osf.io/jfz35/?view_only=4d3861ed0aef461082d88cce5347c1d3

## Footnotes

1 The change in percentage was calculated by the equation [mean_post_—mean_pre_ / mean_pre_].

## Code availability

The codes used in this study were not available to the public.

## Declarations

### Open science statement

This study was pre-registered (for tonal language speakers: (https://osf.io/cqtwf/?view_only=51e9e2bb32774ee986d971ce77a21c40; for non-tonal language speakers: https://osf.io/89en3/?view_only=8f2bccd806f04b85991f2aaf94e212bb).
