# Supplementary material for: Learning fast and accurate absolute pitch judgment in adulthood
Source: Psychon Bull Rev. 2025 Feb 12;32(4):1676–88. doi: 10.3758/s13423-024-02620-2 (PMC12325523; doi:10.3758/s13423-024-02620-2)
Supplement: Supplementary file 1 — Supplementary file1 (DOCX 28 KB) [file 13423_2024_2620_MOESM1_ESM.docx]

Supplementary Information

| Number of Pitches Learned | Response Time Window (ms) |
| --- | --- |
| 1 | 1183 |
| 2 | 1242 |
| 3 | 1305 |
| 4 | 1371 |
| 5 | 1440 |
| 6 | 1513 |
| 7 | 1589 |
| 8 | 1668 |
| 9 | 1751 |
| 10 | 1839 |
| 11 | 1930 |
| 12 | 2028 |

Supplementary Table 1. Response time windows used for training different set sizes of pitches during the AP training. The response time window indicated the requirement during the last no-feedback level that participants needed to pass before an additional pitch was added to the training set.

|  | Pianists | | |  | NonPianists | | |
| --- | --- | --- | --- | --- | --- | --- | --- |
|  | Pre | Post | Post - Pre |  | Pre | Post | Post - Pre |
| Proportion Correct | 0.123 | 0.295 | 0.172 |  | 0.17 | 0.361 | 0.191 |
| Size of Error (Semitones) | 2.7 | 2.06 | -0.64 |  | 2.71 | 1.47 | -1.24 |

Supplementary Table 2. The mean performance and degree of improvement of the participants who had piano as a major instrument (‘Pianists’; N = 8) or not (‘Non-Pianists’; N = 4) for the trained timbre.

***Pre-registration***

The study deviated from the pre-registration in terms of the following. First, we decided to drop the 1^st^ research question about the percentage of participants who could acquire AP in the population since it required a sizable sample. Instead, we focused on the 2^nd^ research question – to test the learnability of AP in adulthood after minimizing the methodological issues in previous studies.

Second, the data for the tonal and non-tonal speakers were analyzed together to increase sample size. This was deemed appropriate because the training protocol they went through were identical.

Third, instead of excluding all participants who failed to complete the 25-hour training over 8 weeks, we decided to include participants who completed 10 hours of training or more and invited them for post-test. This allowed us to examine the AP learning for those who had considerable amount of training comparable to the training durations of many perceptual training studies in the literature (Gauthier et al., 1998; Wong et al., 2009; Wong et al., 2011). This decision was made without having any idea about the final number of participants to be included because the start date of the training varied between participants, and before any post-test data were examined.

Fourth, the response time windows used in this training were longer than those stated in the pre-registration document because pilot data showed that the original time windows were unachievable for many.

Otherwise, the study was conducted as described in the pre-registration documents.

***Prepost Tests (After excluding the two participants that performed considerably better than other participants)***

After excluding these two participants who performed considerably better than other participants (as shown in Figure 2), results of the 2 x 2 Analyses of Variance (ANOVAs) with Prepost (pre-test / post-test) and Timbre (trained / untrained) as within-subject factors remained similar. Similar to the ANOVA reported in the main text, one participant was not included in the RT analyses because no trial was performed correctly during pre-test (Figure 2).

Specifically, there remained a significant main effect of Prepost for for semitone error, *F*(1,9) =12.8, *p* = .006, *η_p_^2^* = .587 and that for proportion correct was marginally significant, *F*(1,9) = 4.91, *p* = .054, *η_p_^2^* = .353, both indicating a higher accuracy after training. The originally marginal main effect of Prepost for correct RT was not significant, *F*(1,8) = 1.79, *p* = .218, *η_p_^2^* = .183. The main effect of Timbre was significant for semitone error, *F*(1,9) = 6.01, *p* = .037, *η_p_^2^* = .401, and for correct RT, *F*(1,8) = 6.39, *p* = .035, *η_p_^2^* = 444, with a reduced error and faster responses for the trained than untrained timbre. The interaction between Prepost and Timbre was significant for proportion correct, *F*(1,9) = 7.15, *p* = .025, *η_p_^2^* = .443, and for semitone error, *F*(1,9) = 7.97, *p* = .020, *η_p_^2^* = .470. Post-hoc Tukey tests (*p <* .05) revealed that proportion correct was higher after training for the trained timbre but not for the untrained timbre, while the semitone error for the trained timbre dropped significantly after training and that for the untrained timbre was marginally significant (*p* = .077).

***Prepost Tests (only for those who completed the 25 hours of training)***

Here, AP performance was examined before and after training only among those who completed the training, i.e., those who have passed all levels of training and those who completed 25 hours of training (as originally planned in the pre-registration; N = 6). Results remained similar to that included all participants who completed the posttests. The differences in results (apart from numerical changes) included: (1) The main effect of Prepost for correct RT changed from a marginal trend to a significant effect here; (2) The significant main effect of Timbre for semitone error no longer reached significance here; (3) The significant interactions between Prepost and Timbre for proportion correct and semitone error became marginal trends here. The detailed results were reported below.

The 2 x 2 Analyses of Variance (ANOVAs) with Prepost (pre-test / post-test) and Timbre (trained / untrained) as within-subject factors showed a main effect of Prepost for proportion correct, *F*(1,5) = 17.3, *p* = .009, *η_p_^2^* = .775, and for semitone error, *F*(1,5) = 21.7, *p* = .006, *η_p_^2^* = .813, both indicating a higher accuracy after training. There was a 165.7% increase in proportion correct (mean_pre_ = .172; mean_post_ = .457) and a 36.8% decrease in semitone error (mean_pre_ = 2.58 semitones; mean_post_ = 1.63 semitones) after training. The main effect of Prepost was also significant for correct RT, *F*(1,4) = 15.9, *p* = .016, *η_p_^2^* = .799, with a 27.1% decrease in correct RT after training (mean_pre_ = 2765ms; mean_post_ = 2017ms). The main effect of Timbre did not reach significance for all three measures (*ps* > .27). The interaction between Prepost and Timbre was a marginally significant trend for proportion correct, *F*(1,5) = 5.45, *p* = .067, *η_p_^2^* = .522, and for semitone error, *F*(1,5) = 4.98, *p* = .076, *η_p_^2^* = .499. Post-hoc Tukey tests (*p <* .05) revealed that the proportion correct increased after training for the trained timbre, but that for the untrained timbre was only marginally significant (*p* = .056). The semitone error dropped for both timbres but more so for the trained timbre.
